# Attention-Deficit/Hyperactivity Disorder in Anterior Cruciate Ligament Reconstruction Patients ≤25 Years of Age: A National Database Study of Prevalence and Outcomes

**DOI:** 10.31486/toj.26.0021

**Published:** 2026

**Authors:** David Lutati, Jane C. Brennan, Matthew Peterman, Andrea H. Johnson, James York, Benjamin M. Petre, Daniel E. Redziniak, Justin J. Turcotte

**Affiliations:** Department of Orthopedics, Luminis Health, Anne Arundel Medical Center, Annapolis, MD

**Keywords:** *Adolescent*, *adult*, *anterior cruciate ligament injuries*, *attention deficit disorder with hyperactivity*, *comorbidity*, *mental disorders*, *patient outcome assessment*, *postoperative complications*

## Abstract

**Background:**

A growing body of research indicates that psychiatric conditions put patients at risk for worse outcomes after anterior cruciate ligament (ACL) reconstruction. However, attention-deficit/hyperactivity disorder (ADHD) is an understudied comorbidity in patients undergoing ACL reconstruction.

**Methods:**

Using the PearlDiver national database, we conducted a retrospective review of 9,352 patients ≤25 years who underwent isolated ACL reconstruction during the period 2010 to 2023. We compared demographics, comorbidities, and outcomes between patients with and without an ADHD diagnosis before and after 1:3 propensity score matching. We also performed a subgroup analysis of ADHD patients who were on and off medication.

**Results:**

On average, patients with ADHD were younger than patients without ADHD (*P*<0.001); were significantly more likely to be male; and had increased rates of obesity, alcohol use disorders, anxiety disorders/depression, tobacco use, and developmental disorders (all *P*<0.001). Postoperatively, we found no differences in rates of 90-day readmission or in 2-year prolonged opioid use, repeat ACL reconstruction, other knee surgery, or stiffness either before or after propensity score matching. Among the patients with ADHD, 420 (67.9%) filled a prescription for ADHD medication in the 90 days prior to ACL reconstruction. No significant differences were detected in 90-day or 2-year outcomes between patients who used ADHD medication and those who did not.

**Conclusion:**

In this retrospective database study, 6.6% of patients ≤25 years who underwent ACL reconstruction had a diagnosis of ADHD prior to surgery. Neither a diagnosis of ADHD nor ADHD medication use was associated with worse outcomes after ACL reconstruction. Given the high prevalence of ADHD, further study of its effects on outcomes of ACL reconstruction and other orthopedic conditions is needed.

## INTRODUCTION

Anterior cruciate ligament (ACL) reconstruction is widely considered the gold standard for restoring knee function and stability after an ACL tear.^1,2^ Aside from effectively improving patient-reported outcomes and function,^3,4^ ACL reconstruction also has a low risk profile for major postoperative adverse events, such as infection requiring reoperation, pulmonary embolism, and neurovascular injury.^5^ While ACL reconstruction is generally considered a safe and effective procedure, potential postoperative complications include stiffness, anterior knee pain, secondary meniscal injuries, graft failure, and infection.^6,7^

A growing body of research indicates that psychiatric conditions can put patients at risk for worse outcomes after ACL reconstruction.^8-12^ Patients with mental health disorders have been shown to experience higher rates of postoperative infection, lower extremity neuropathy, pain, and knee stiffness.^10^ Depression and anxiety are the most commonly studied psychiatric conditions in patients undergoing ACL reconstruction and have consistently been shown to play a role in poorer functional outcomes after surgery.^8,11,12^ Notably, attention-deficit/hyperactivity disorder (ADHD) is a lesser studied comorbidity in patients undergoing ACL reconstruction. Compared to individuals without ADHD, patients with ADHD have been shown to have lower life expectancies with higher rates of both unnatural and natural causes of death.^13,14^ In addition to lower life expectancies, ADHD patients report worse functioning in education, work, and social settings than individuals without the condition.^15,16^ However, a paucity of studies has examined the effects of ADHD on orthopedic surgery outcomes, as <1% of studies of mental health in orthopedic surgery evaluated ADHD from 2001 to 2022.^17^

In this retrospective database study, we aimed to evaluate the prevalence of ADHD and accompanying comorbidities in patients undergoing ACL reconstruction, compare postoperative outcomes between patients with and without an ADHD diagnosis, and assess if pharmacologic treatment improved postoperative outcomes in the ADHD population. We hypothesized that patients with ADHD undergoing ACL reconstruction would have higher rates of comorbidities and poorer postoperative outcomes compared to patients without ADHD. We also hypothesized that treatment would play a protective role in patients with ADHD undergoing ACL reconstruction.

## METHODS

As a retrospective review of a deidentified database, this study was deemed exempt by the institutional review board.

### Data Source

We retrospectively analyzed the PearlDiver Mariner 170 dataset (PearlDiver Technologies, Inc).^18^ The database contains claims records from more than 170 million patients across all payers, including commercial insurance, Medicare, Medicaid, and self-pay. Data are searchable by predefined cohorts and by *International Classification of Diseases, Ninth Revision* (ICD-9), *International Classification of Diseases, Tenth Revision* (ICD-10), and Current Procedural Terminology (CPT) codes.

### Study Population

All 9,352 patients included in this study were ≤25 years and underwent primary ACL reconstruction between 2010 and 2023. Patients were active in the database (ie, had claims available) for at least 2 years prior to ACL reconstruction and 2 years postoperatively. Patients were grouped by whether they were or were not diagnosed with ADHD prior to ACL reconstruction. Patients with concomitant medial collateral ligament, posterior cruciate ligament, or meniscus repairs were excluded from this study. Additionally, patients with a history of birth trauma, brain/nervous system cancer, coma/stupor/brain damage, behavioral or emotional disorders diagnosed before adulthood, cognitive disorders, encephalitis, intracranial injury, meningitis, nervous system anomalies, other nervous system conditions or disorders, and other central nervous system conditions (eg, poliomyelitis, Creutzfeldt-Jakob disease, and other viral infections) were excluded from this study. Inclusion and exclusion criteria definitions are listed in Appendix 1.

### Independent Variables

Demographics and comorbidities (based on ICD-9/10 codes) of interest were age, sex, Charlson Comorbidity Index (CCI) score, alcohol use disorders, obesity, tobacco use, anxiety disorders/depression, and developmental disorders. Patient age and sex were obtained from predefined demographic variables in the PearlDiver database. Age corresponded to the patient's age at the time of the index ACL reconstruction procedure, and sex was recorded as a binary categorical variable (male/female). Comorbidity burden was assessed using the CCI score, which was extracted as a precomputed variable in PearlDiver. The CCI score is generated within the database using ICD-9 and ICD-10 diagnosis codes and is calculated according to the validated Charlson weighting algorithm. Cormorbidity definitions are provided in Appendix 2.

### Outcome Measures and Medication Use

Readmission, defined as an inpatient encounter within 90 days of discharge, was assessed at 90 days postoperatively. Repeat ACL reconstruction, other knee surgery, and stiffness were assessed at 2 years. Opioid use was defined as prolonged use continuing at 2 years postoperatively. Outcome measures definitions are provided in Appendix 3.

ADHD medication use was identified using outpatient pharmacy claims in the PearlDiver database. Medications were extracted using standardized drug identifiers (eg, DRUG-METHYLPHENIDATE) and grouped into clinically relevant categories: stimulants, nonstimulants, agonists, and other drugs. The other drugs category includes agents such as norepinephrine and dopamine reuptake inhibitors, serotonin and norepinephrine reuptake inhibitors, anticonvulsants (eg, divalproex), lithium, antipsychotics (eg, olanzapine, risperidone), selective serotonin reuptake inhibitors (eg, sertraline, fluoxetine, paroxetine), clonazepam, hydroxyzine, and trazodone. Medication definitions are provided in Appendix 3.

### Statistical Analysis

Univariate analyses (chi-square and independent samples *t* tests) were used to compare demographics, comorbidities, and outcomes between groups. Patients without ADHD were nearest neighbor propensity score matched 3:1 to patients with ADHD for all independent variables assessed. Differences in outcomes were assessed using univariate analysis between propensity score matched groups. Differences are presented as occurrence rates (n, %) and risk ratios with 95% confidence intervals. Patients with ADHD were further split into those who filled an ADHD medication prescription in the 90 days prior to ACL reconstruction and those who did not. Medication exposure was defined as at least 1 prescription fill within 90 days prior to the index ACL reconstruction procedure. Differences in outcomes between patients on medication and those not on medication were assessed using univariate analysis. All statistical analysis was performed within the PearlDiver platform using R (Posit Software, PBC). Statistical significance was assessed at *P*<0.05.

### Power Analysis

Prior to study initiation, an a priori power analysis was performed to assess sample size requirements. Based on a 3:1 cohort ratio, 306 ADHD patients and 918 non-ADHD patients were deemed necessary to detect 5% differences in categorical outcomes at 80% power using chi-square testing.

## RESULTS

Overall, 9,352 patients who underwent ACL reconstruction were included in this study, 6.6% of whom (n=619) were diagnosed with ADHD prior to surgery. On average, patients with ADHD were 4.5 years younger at the time of ACL reconstruction than those without ADHD (13.6 ± 4.0 years vs 18.1 ± 3.2 years; *P*<0.001). Further, the proportion of male patients was significantly higher in the ADHD group than the non-ADHD group (62.7% vs 41.9%; *P*<0.001). Additionally, those with ADHD had increased rates of alcohol use disorders (3.1% vs 1.0%; *P*<0.001), obesity (9.7% vs 6.1%; *P*<0.001), tobacco use (6.0% vs 2.2%; *P*<0.001), anxiety disorders/depression (31.5% vs 10.2%; *P*<0.001), and developmental disorders (4.0% vs 0.4%; *P*<0.001). Overall, the comorbidity burden, as measured by the CCI score, was low in both groups (<0.5) and did not differ significantly (Table 1).

**Table 1.** Demographics and Comorbidities Prior to Propensity Score Matching

Variable	Non-ADHD Group, n=8,733	ADHD Group, n=619	*P* Value
Demographics			
Age, years, mean ± SD	18.1 ± 3.2	13.6 ± 4.0	**<0.001**
Charlson Comorbidity Index score, mean ± SD	0.2 ± 0.4	0.1 ± 0.4	0.240
Sex			**<0.001**
Female	5,078 (58.1)	231 (37.3)	
Male	3,655 (41.9)	388 (62.7)	
Comorbidity			
Alcohol use disorders	83 (1.0)	19 (3.1)	**<0.001**
Obesity	531 (6.1)	60 (9.7)	**<0.001**
Tobacco use	192 (2.2)	37 (6.0)	**<0.001**
Anxiety disorders/depression	893 (10.2)	195 (31.5)	**<0.001**
Developmental disorders	34 (0.4)	25 (4.0)	**<0.001**

Notes: Data are reported as n (%) unless otherwise indicated. *P* values <0.05 are shown in bold.

ADHD, attention-deficit/hyperactivity disorder.

Postoperatively, no differences were found in rates of 90-day readmission (risk ratio [RR] 0.35, 95% CI 0.14-0.92; *P*=0.064) or in 2-year repeat ACL reconstruction (RR 0.93, 95% CI 0.59-1.48; *P*=0.863), other knee surgery (RR 0.97, 95% CI 0.72-1.31; *P*=0.912), stiffness (RR 1.03, 95% CI 0.84-1.25; *P*=0.844), or prolonged opioid use (RR 0.91, 95% CI 0.80-1.04; *P*=0.176) prior to propensity score matching (Table 2).

**Table 2.** Outcomes at 90 Days and 2 Years Prior to Propensity Score Matching

Time Point/Outcome	Non-ADHD Group, n=8,733	ADHD Group, n=619	Risk Ratio (95% CI)	*P* Value
90-day time point				
Readmission	25 (0.3)	5 (0.8)	0.35 (0.14-0.92)	0.064
2-year time point				
Repeat ACL reconstruction	250 (2.9)	19 (3.1)	0.93 (0.59-1.48)	0.863
Other knee surgery	575 (6.6)	42 (6.8)	0.97 (0.72-1.31)	0.912
Stiffness	1,317 (15.1)	91 (14.7)	1.03 (0.84-1.25)	0.844
Opioid use	2,287 (26.2)	178 (28.8)	0.91 (0.80-1.04)	0.176

Note: Data are reported as n (%).

ACL, anterior cruciate ligament; ADHD, attention-deficit/hyperactivity disorder; CI confidence interval.

After propensity score matching, no differences were detected in age, sex, or rates of 4 of the 5 individual comorbidities. However, after matching, patients with ADHD remained more likely to have developmental disorders (4.0% vs 1.8%; *P*=0.003) (Table 3). Postoperatively, after propensity score matching, no significant differences were found in rates of 90-day readmission (RR 1.67, 95% CI 0.56-4.95; *P*=0.536) or in 2-year repeat ACL reconstruction (RR 1.10, 95% CI 0.65-1.84; *P*=0.835), other knee surgery (RR 1.10, 95% CI 0.78-1.54; *P*=0.668), stiffness (RR 0.96, 95% CI 0.77-1.20; *P*=0.771), or prolonged opioid use (RR 1.07, 95% CI 0.93-1.24; *P*=0.390) (Table 4).

**Table 3.** Demographics and Comorbidities After Propensity Score Matching

Variable	Non-ADHD Group, n=1,857	ADHD Group, n=619	*P* Value
Demographics			
Age, years, mean ± SD	18.1 ±3.4	18.0 ± 3.3	0.633
Charlson Comorbidity Index score, mean ± SD	0.24 ± 0.51	0.25 ± 0.50	0.584
Sex			0.583
Female	718 (38.7)	231 (37.3)	
Male	1,139 (61.3)	388 (62.7)	
Comorbidity			
Alcohol use disorders	43 (2.3)	19 (3.1)	0.373
Obesity	220 (11.8)	60 (9.7)	0.164
Tobacco use	102 (5.5)	37 (6.0)	0.724
Anxiety disorders/depression	604 (32.5)	195 (31.5)	0.673
Developmental disorders	34 (1.8)	25 (4.0)	**0.003**

Notes: Data are reported as n (%) unless otherwise indicated. *P* values <0.05 are shown in bold.

ADHD, attention-deficit/hyperactivity disorder.

**Table 4.** Outcomes at 90 Days and 2 Years After Propensity Score Matching

Time Point/Outcome	Non-ADHD Group, n=1,857	ADHD Group, n=619	Risk Ratio (95% CI)	*P* Value
90-day time point				
Readmission	9 (0.5)	5 (0.8)	1.67 (0.56-4.95)	0.536
2-year time point				
Repeat ACL reconstruction	52 (2.8)	19 (3.1)	1.10 (0.65-1.84)	0.835
Other knee surgery	115 (6.2)	42 (6.8)	1.10 (0.78-1.54)	0.668
Stiffness	284 (15.3)	91 (14.7)	0.96 (0.77-1.20)	0.771
Opioid use	499 (26.9)	178 (28.8)	1.07 (0.93-1.24)	0.390

Note: Data are reported as n (%).

ACL, anterior cruciate ligament; ADHD, attention-deficit/hyperactivity disorder; CI confidence interval.

Among the 619 patients with ADHD, 420 (67.9%) filled a prescription for ADHD medication in the 90 days prior to ACL reconstruction. We found no statistically significant differences in any of the outcomes between those who used ADHD medication and those who did not (Table 5): 90-day readmission (RR 0.71, 95% CI 0.12-4.22; *P*=1), 2-year repeat ACL reconstruction (RR 1.03, 95% CI 0.40-2.66; *P*=1), 2-year other knee surgery (RR 1.06, 95% CI 0.56-1.99; *P*=0.999), 2-year stiffness (RR 0.92, 95% CI 0.62-1.37; *P*=0.762), or prolonged opioid use (RR 1.15, 95% CI 0.87-1.51; *P*=0.369).

**Table 5.** Outcomes at 90 Days and 2 Years by Medication Use in Patients With ADHD, n=619

Time Point/Outcome	No ADHD Medication Group, n=199	ADHD Medication Group, n=420	Risk Ratio (95% CI)	*P* Value
90-day time point				
Readmission	2 (1.0)	3 (0.7)	0.71 (0.12-4.22)	1
2-year time point				
Repeat ACL reconstruction	6 (3.0)	13 (3.1)	1.03 (0.40-2.66)	1
Other knee surgery	13 (6.5)	29 (6.9)	1.06 (0.56-1.99)	0.999
Stiffness	31 (15.6)	60 (14.3)	0.92 (0.62-1.37)	0.762
Opioid use	52 (26.1)	126 (30.0)	1.15 (0.87-1.51)	0.369

Note: Data are reported as n (%).

ACL, anterior cruciate ligament; ADHD, attention-deficit/hyperactivity disorder; CI, confidence interval.

## DISCUSSION

In the current study, we observed no significant differences in postoperative outcomes between patients with and without ADHD or between patients with ADHD who were on medication and those who were not, findings that are contrary to our initial hypotheses. We saw similar outcomes in both the unmatched and propensity score matched analyses. While ADHD was not associated with increased complications after ACL reconstruction, ADHD was associated with differences in patient demographics of age and sex and in rates of comorbidities including alcohol use disorders, tobacco use, and obesity, findings that are partially in line with our initial hypotheses. In addition to these lifestyle risk factors, patients with ADHD were significantly more likely to have a concurrent anxiety disorders/depression diagnosis or developmental disorders.

Our finding that a greater proportion (62.7% vs 41.9%) of ACL reconstruction patients with ADHD were male was expected, given the higher rates of ADHD diagnosis in males in the general population. According to 2022 national survey data, 14.7% of males and 8.1% of female children have received an ADHD diagnosis.^19^ While ADHD has not been previously associated with increased risk of ACL rupture specifically, ADHD has consistently been shown to increase the risk of other unintentional injuries in both pediatric and adult populations.^20,21^ Given the hallmark symptoms of pediatric ADHD, such as impulsivity, inattention, and increased risk-taking,^22,23^ these factors possibly contributed to the age differences observed, with ADHD patients experiencing ACL injuries requiring surgery at younger ages. However, direct study of the incidence of ACL rupture and surgery in the ADHD and non-ADHD populations is required to confirm this hypothesis.

As seen from our results, patients with ADHD undergoing ACL reconstruction were significantly more likely to be preexisting tobacco users or have alcohol use disorders. ADHD is associated with higher rates of tobacco or e-cigarette use, as well as increased rates of alcohol use disorders,^24-27^ and substance use is known to increase the rate of postoperative complications after ACL reconstruction.^28-31^ In a retrospective database study, Cancienne et al examined the relationship between tobacco use and rates of postoperative complications after ACL reconstruction and found that tobacco users were more likely to experience venous thromboembolism, surgical site infection, or subsequent ACL reconstruction.^31^ Eliasen et al examined the effect of preoperative alcohol use on postoperative outcomes across a variety of orthopedic, gastrointestinal, and general surgery procedures.^30^ Their meta-analysis demonstrated that preoperative alcohol use was associated with higher rates of infections, wound complications, and pulmonary complications.^30^ While ADHD was not associated with higher rates of postoperative complications in our study, the strong association of ADHD with substance use must be considered for patients undergoing ACL reconstruction. Based on our findings, surgeons should comprehensively screen patients with ADHD for other lifestyle risk factors that may be modifiable prior to surgery.

Among the patients undergoing ACL reconstruction in our study, those with ADHD had a higher rate of preoperative anxiety disorders/depression. The link between ADHD and concomitant mental health conditions is often attributed to issues with executive functioning, emotional regulation, and response inhibition.^32-34^ Furthermore, mental health disorders are known to be associated with worse functional and patient-reported outcomes after ACL reconstruction.^8-12^ In a retrospective database study, Kesaria et al examined the relationship between mental health disorders, including anxiety or depression, and postoperative complications after ACL reconstruction.^10^ Their analysis showed that patients with preoperative mental health disorders had higher rates of postoperative infection, lower limb neuropathy, pain, knee stiffness, postoperative opioid use, and patella fracture compared to patients without diagnosed mental health disorders. Similarly, Hinton et al found that pediatric patients with a diagnosis of depression prior to ACL reconstruction were more likely to use both opioid and nonopioid analgesics, experience higher rates of joint stiffness and effusion, and undergo reoperation within 6 months postoperatively.^9^ While our findings indicate that ADHD is not independently associated with poorer outcomes after ACL reconstruction, the strong association of ADHD with depression and anxiety suggests that a multidisciplinary approach incorporating early mental health screening and support may be warranted in this population.

Despite the paucity of studies evaluating the effect of ADHD on orthopedic surgery outcomes, the potential implications have been assessed in other surgical populations. Zwemer et al found that patients with ADHD and another mental disorder were more likely to present with higher injury severity scores and to experience longer hospital lengths of stay after trauma surgery compared to patients with ADHD only.^35^ Stenberg et al found that patients with ADHD undergoing bariatric or metabolic surgery were more likely to experience a postoperative complication, engage in self-harm, and engage in substance abuse compared to patients without ADHD.^36^ The reasons why patients with ADHD have worse outcomes after trauma and bariatric surgery but not after ACL reconstruction are unclear. One possibility is that ACL reconstruction has a relatively stable and rigid protocol following surgery, which may provide adequate structure to patients with worse baseline executive function. However further research is warranted to evaluate whether these findings are replicable and consistent in other elective orthopedic procedures.

Contrary to our hypotheses, ADHD had no association with worse outcomes after ACL reconstruction in this study, and medication had no beneficial effect in the postoperative period. Multiple studies have reported better outcomes in patients with ADHD who take medication, including lower rates of self-harm, substance abuse, and better professional or academic functioning.^37-39^ Although the potential effects of ADHD medication on long-term surgical outcomes have been sparsely studied, Yoon et al examined the topic in a large retrospective database study.^40^ When comparing matched cohorts of 337,147 ADHD patients with and without stimulant prescriptions who underwent surgery, medication had mixed effects on various surgical outcomes. Medication showed a protective effect against 3 of 12 complications assessed (including infection), while increasing the risk of 4 complication types (including orthopedic prosthesis-related complications). Given these mixed results and the negative results of our study, further research into the potential effects of ADHD medication on ACL reconstruction patients specifically and surgical patients generally is warranted.

This study has limitations. Database studies are limited by the accuracy of coded data, and this limitation is of particular importance in the current study, as we relied on diagnosis codes to identify ADHD patients. Consequently, patients with undiagnosed ADHD could potentially have been included in the control group. Additionally, given the reliance on coded data, we were unable to gauge the severity of patients’ ADHD, which can range from mild to severe. Furthermore, we were unable to assess other patient-specific behaviors such as medication adherence and adherence to rehabilitation regimens. Selection bias is likely present in the study, although we attempted to mitigate the potential effect through the use of extensive exclusion criteria and propensity score matching. However, unmeasured confounding factors, such as patient-specific pathophysiology, surgical technique, and socioeconomic status, could have contributed to the results. Additionally, we were unable to assess critical postoperative outcomes such as patient-reported outcome measures and functional outcomes that may be affected by mental health disorders such as ADHD or comorbid depression and anxiety. Further, this study used a relatively short follow-up duration of 2 years postoperatively; therefore, studies with longer follow-up periods are warranted. With regard to statistical analysis, while an a priori power analysis was performed, differences in rates of categorical outcomes observed in the study did not reach the 5% threshold necessary to meet the 80% power target. Therefore, all negative results are potentially attributable to type II error and should be interpreted with caution. Finally, this study intentionally focused on patients ≤25 years given the higher rates of ADHD diagnosis in younger populations, so our findings cannot be extrapolated to older patients undergoing ACL reconstruction. Replication of this study in patients >25 years is warranted to evaluate the effects of ADHD in this population. While this study is one of the first to evaluate the outcomes of ACL reconstruction in patients with and without ADHD, future studies using granular clinical data, patient-reported outcome measures, and longer follow-up are needed.

## CONCLUSION

In this study, 6.6% of patients ages ≤25 years who underwent ACL reconstruction had a diagnosis of ADHD prior to surgery. In comparison to patients without an ADHD diagnosis, the patients with ADHD were slightly younger; included a greater proportion of male patients; and had higher rates of alcohol use disorders, obesity, tobacco use, anxiety disorders/depression, and developmental disorders. A diagnosis of ADHD and ADHD medication use were not associated with differential outcomes after ACL reconstruction at 90 days and 2 years postoperatively. Given the high prevalence of ADHD, further study of the effects of ADHD on outcomes of ACL reconstruction and other orthopedic procedures is needed.

## Notes

*This article meets the Accreditation Council for Graduate Medical Education and the American Board of Medical Specialties Maintenance of Certification competencies for Patient Care, Medical Knowledge, and Practice-Based Learning and Improvement.*

## Appendix 1. Study Inclusion and Exclusion Criteria Definitions

**Table:**

Study Criteria	*International Classification of Diseases, Ninth Revision* (ICD-9), *International Classification of Diseases, Tenth Revision* (ICD-10), and Current Procedural Terminology (CPT) Codes
Inclusion^a^	
Anterior cruciate ligament reconstruction	CPT-29888
Attention-deficit/hyperactivity disorder	ICD-10-D-F900, ICD-10-D-F901, ICD-10-D-F902, ICD-10-D-F908, ICD-10-D-F909, ICD-9-D-31400, ICD-9-D-31401, ICD-9-D-3141, ICD-9-D-3142, ICD-9-D-3148, ICD-9-D-3149
Exclusion	
Concomitant medial collateral ligament/posterior cruciate ligament injury or repair	CPT-27407, CPT-27427, CPT-27428, CPT-27429, CPT-27405, CPT-29889, ICD-10-D-M23621, ICD-10-D-M23622, ICD-10-D-M23629, ICD-10-D-S83521A, ICD-10-D-S83521D, ICD-10-D-S83521S, ICD-10-D-S83522A, ICD-10-D-S83522D, ICD-10-D-S83522S, ICD-10-D-S83529A, ICD-10-D-S83529D, ICD-10-D-S83529S, ICD-9-D-8440, ICD-9-D-8441
Concomitant meniscus repair	CPT-29880, CPT-29881, CPT-29882, CPT-29883
Birth trauma	ICD-10-D-P100, ICD-10-D-P101, ICD-10-D-P102, ICD-10-D-P103, ICD-10-D-P104, ICD-10-D-P108, ICD-10-D-P109, ICD-10-D-P110, ICD-10-D-P111, ICD-10-D-P112, ICD-10-D-P113, ICD-10-D-P114, ICD-10-D-P115, ICD-10-D-P119, ICD-10-D-P120, ICD-10-D-P121, ICD-10-D-P122, ICD-10-D-P123, ICD-10-D-P124, ICD-10-D-P1281, ICD-10-D-P1289, ICD-10-D-P129, ICD-10-D-P130, ICD-10-D-P131, ICD-10-D-P132, ICD-10-D-P133, ICD-10-D-P134, ICD-10-D-P138, ICD-10-D-P139, ICD-10-D-P140, ICD-10-D-P141, ICD-10-D-P142, ICD-10-D-P143, ICD-10-D-P148, ICD-10-D-P149, ICD-10-D-P150, ICD-10-D-P151, ICD-10-D-P152, ICD-10-D-P153, ICD-10-D-P154, ICD-10-D-P155, ICD-10-D-P156, ICD-10-D-P158, ICD-10-D-P159, ICD-9-D-7670, ICD-9-D-76711, ICD-9-D-76719, ICD-9-D-7672, ICD-9-D-7673, ICD-9-D-7674, ICD-9-D-7675, ICD-9-D-7676, ICD-9-D-7677, ICD-9-D-7678, ICD-9-D-7679, ICD-9-D-77210, ICD-9-D-7722
Brain/nervous system cancer	ICD-10-D-C470, ICD-10-D-C4710, ICD-10-D-C4711, ICD-10-D-C4712, ICD-10-D-C4720, ICD-10-D-C4721, ICD-10-D-C4722, ICD-10-D-C473, ICD-10-D-C474, ICD-10-D-C475, ICD-10-D-C476, ICD-10-D-C478, ICD-10-D-C479, ICD-10-D-C700, ICD-10-D-C701, ICD-10-D-C709, ICD-10-D-C710, ICD-10-D-C711, ICD-10-D-C712, ICD-10-D-C713, ICD-10-D-C714, ICD-10-D-C715, ICD-10-D-C716, ICD-10-D-C717, ICD-10-D-C718, ICD-10-D-C719, ICD-10-D-C720, ICD-10-D-C721, ICD-10-D-C7220, ICD-10-D-C7221, ICD-10-D-C7222, ICD-10-D-C7230, ICD-10-D-C7231, ICD-10-D-C7232, ICD-10-D-C7240, ICD-10-D-C7241, ICD-10-D-C7242, ICD-10-D-C7250, ICD-10-D-C7259, ICD-10-D-C729, ICD-10-D-Z85841, ICD-10-D-Z85848, ICD-9-D-1910, ICD-9-D-1911, ICD-9-D-1912, ICD-9-D-1913, ICD-9-D-1914, ICD-9-D-1915, ICD-9-D-1916, ICD-9-D-1917, ICD-9-D-1918, ICD-9-D-1919, ICD-9-D-1920, ICD-9-D-1921, ICD-9-D-1922, ICD-9-D-1923, ICD-9-D-1928, ICD-9-D-V1085, ICD-9-D-V1086
Coma/stupor/brain damage	ICD-10-D-G931, ICD-10-D-R400, ICD-10-D-R401, ICD-10-D-R4020, ICD-10-D-R402110, ICD-10-D-R402111, ICD-10-D-R402112, ICD-10-D-R402113, ICD-10-D-R402114, ICD-10-D-R402120, ICD-10-D-R402121, ICD-10-D-R402122, ICD-10-D-R402123, ICD-10-D-R402124, ICD-10-D-R402130, ICD-10-D-R402131, ICD-10-D-R402132, ICD-10-D-R402133, ICD-10-D-R402134, ICD-10-D-R402140, ICD-10-D-R402141, ICD-10-D-R402142, ICD-10-D-R402143, ICD-10-D-R402144, ICD-10-D-R402210, ICD-10-D-R402211, ICD-10-D-R402212, ICD-10-D-R402213, ICD-10-D-R402214, ICD-10-D-R402220, ICD-10-D-R402221, ICD-10-D-R402222, ICD-10-D-R402223, ICD-10-D-R402224, ICD-10-D-R402230, ICD-10-D-R402231, ICD-10-D-R402232, ICD-10-D-R402233, ICD-10-D-R402234, ICD-10-D-R402240, ICD-10-D-R402241, ICD-10-D-R402242, ICD-10-D-R402243, ICD-10-D-R402244, ICD-10-D-R402250, ICD-10-D-R402251, ICD-10-D-R402252, ICD-10-D-R402253, ICD-10-D-R402254, ICD-10-D-R402310, ICD-10-D-R402311, ICD-10-D-R402312, ICD-10-D-R402313, ICD-10-D-R402314, ICD-10-D-R402320, ICD-10-D-R402321, ICD-10-D-R402322, ICD-10-D-R402323, ICD-10-D-R402324, ICD-10-D-R402330, ICD-10-D-R402331, ICD-10-D-R402332, ICD-10-D-R402333, ICD-10-D-R402334, ICD-10-D-R402340, ICD-10-D-R402341, ICD-10-D-R402342, ICD-10-D-R402343, ICD-10-D-R402344, ICD-10-D-R402350, ICD-10-D-R402351, ICD-10-D-R402352, ICD-10-D-R402353, ICD-10-D-R402354, ICD-10-D-R402360, ICD-10-D-R402361, ICD-10-D-R402362, ICD-10-D-R402363, ICD-10-D-R402364, ICD-10-D-R40241, ICD-10-D-R402410, ICD-10-D-R402411, ICD-10-D-R402412, ICD-10-D-R402413, ICD-10-D-R402414, ICD-10-D-R40242, ICD-10-D-R402420, ICD-10-D-R402421, ICD-10-D-R402422, ICD-10-D-R402423, ICD-10-D-R402424, ICD-10-D-R40243, ICD-10-D-R402430, ICD-10-D-R402431, ICD-10-D-R402432, ICD-10-D-R402433, ICD-10-D-R402434, ICD-10-D-R40244, ICD-10-D-R402440, ICD-10-D-R402441, ICD-10-D-R402442, ICD-10-D-R402443, ICD-10-D-R402444, ICD-10-D-R403, ICD-9-D-3481, ICD-9-D-78003, ICD-9-D-78009
Behavioral or emotional disorders diagnosed before adulthood	ICD-10-D-F642, ICD-10-D-F840, ICD-10-D-F842, ICD-10-D-F843, ICD-10-D-F845, ICD-10-D-F848, ICD-10-D-F849, ICD-10-D-F930, ICD-10-D-F938, ICD-10-D-F939, ICD-10-D-F940, ICD-10-D-F941, ICD-10-D-F942, ICD-10-D-F948, ICD-10-D-F949, ICD-10-D-F950, ICD-10-D-F951, ICD-10-D-F952, ICD-10-D-F958, ICD-10-D-F959, ICD-10-D-F980, ICD-10-D-F981, ICD-10-D-F9821, ICD-10-D-F9829, ICD-10-D-F983, ICD-10-D-F984, ICD-10-D-F988, ICD-10-D-F989, ICD-9-D-29900, ICD-9-D-29910, ICD-9-D-29980, ICD-9-D-29990, ICD-9-D-3026, ICD-9-D-30720, ICD-9-D-30721, ICD-9-D-30722, ICD-9-D-30723, ICD-9-D-3073, ICD-9-D-30752, ICD-9-D-30753, ICD-9-D-30759, ICD-9-D-3076, ICD-9-D-3077, ICD-9-D-30921, ICD-9-D-3130, ICD-9-D-31323, ICD-9-D-31389, ICD-9-D-3139
Cognitive disorders	ICD-10-D-F0150, ICD-10-D-F0151, ICD-10-D-F0280, ICD-10-D-F0281, ICD-10-D-F0390, ICD-10-D-F0391, ICD-10-D-F04, ICD-10-D-F05, ICD-10-D-F070, ICD-10-D-F0781, ICD-10-D-F0789, ICD-10-D-F079, ICD-10-D-F09, ICD-10-D-F482, ICD-10-D-G300, ICD-10-D-G301, ICD-10-D-G308, ICD-10-D-G309, ICD-10-D-G3101, ICD-10-D-G3109, ICD-10-D-G311, ICD-10-D-G3183, ICD-10-D-R4181, ICD-10-D-R54, ICD-9-D-2900, ICD-9-D-29040, ICD-9-D-29041, ICD-9-D-2930, ICD-9-D-2940, ICD-9-D-29410, ICD-9-D-29411, ICD-9-D-29421, ICD-9-D-3100, ICD-9-D-3102, ICD-9-D-31081, ICD-9-D-31089, ICD-9-D-3109, ICD-9-D-3310, ICD-9-D-33111, ICD-9-D-33119, ICD-9-D-3312, ICD-9-D-33182, ICD-9-D-797
Encephalitis	ICD-10-D-A3212, ICD-10-D-A3981, ICD-10-D-A4282, ICD-10-D-A811, ICD-10-D-A830, ICD-10-D-A831, ICD-10-D-A832, ICD-10-D-A833, ICD-10-D-A834, ICD-10-D-A835, ICD-10-D-A836, ICD-10-D-A838, ICD-10-D-A839, ICD-10-D-A840, ICD-10-D-A841, ICD-10-D-A848, ICD-10-D-A849, ICD-10-D-A850, ICD-10-D-A851, ICD-10-D-A852, ICD-10-D-A858, ICD-10-D-A86, ICD-10-D-A89, ICD-10-D-A922, ICD-10-D-A9231, ICD-10-D-B004, ICD-10-D-B0111, ICD-10-D-B020, ICD-10-D-B050, ICD-10-D-B0601, ICD-10-D-B1001, ICD-10-D-B1009, ICD-10-D-B262, ICD-10-D-B4081, ICD-10-D-B5742, ICD-10-D-B582, ICD-10-D-B6011, ICD-10-D-B941, ICD-10-D-G0400, ICD-10-D-G0401, ICD-10-D-G0402, ICD-10-D-G042, ICD-10-D-G0430, ICD-10-D-G0431, ICD-10-D-G0432, ICD-10-D-G0439, ICD-10-D-G0481, ICD-10-D-G0489, ICD-10-D-G0490, ICD-10-D-G0491, ICD-10-D-G053, ICD-10-D-G054, ICD-9-D-0361, ICD-9-D-0462, ICD-9-D-0498, ICD-9-D-0499, ICD-9-D-0520, ICD-9-D-05319, ICD-9-D-0543, ICD-9-D-0550, ICD-9-D-05601, ICD-9-D-05821, ICD-9-D-05829, ICD-9-D-0620, ICD-9-D-0621, ICD-9-D-0622, ICD-9-D-0623, ICD-9-D-0624, ICD-9-D-0625, ICD-9-D-0628, ICD-9-D-0629, ICD-9-D-0630, ICD-9-D-0631, ICD-9-D-0632, ICD-9-D-0639, ICD-9-D-064, ICD-9-D-0662, ICD-9-D-06641, ICD-9-D-0722, ICD-9-D-1390, ICD-9-D-3209, ICD-9-D-32341, ICD-9-D-32342, ICD-9-D-32351, ICD-9-D-32361, ICD-9-D-32362, ICD-9-D-32381, ICD-9-D-32382, ICD-9-D-3239
Intracranial injury	ICD-10-D-S060 × 0A, ICD-10-D-S060 × 0D, ICD-10-D-S060 × 0S, ICD-10-D-S060 × 1A, ICD-10-D-S060 × 1D, ICD-10-D-S060 × 1S, ICD-10-D-S060 × 2A, ICD-10-D-S060 × 2D, ICD-10-D-S060 × 2S, ICD-10-D-S060 × 3A, ICD-10-D-S060 × 3D, ICD-10-D-S060 × 3S, ICD-10-D-S060 × 4A, ICD-10-D-S060 × 4D, ICD-10-D-S060 × 4S, ICD-10-D-S060 × 5A, ICD-10-D-S060 × 5D, ICD-10-D-S060 × 5S, ICD-10-D-S060 × 6A, ICD-10-D-S060 × 6D, ICD-10-D-S060 × 6S, ICD-10-D-S060 × 7A, ICD-10-D-S060 × 7D, ICD-10-D-S060 × 7S, ICD-10-D-S060 × 8A, ICD-10-D-S060 × 8D, ICD-10-D-S060 × 8S, ICD-10-D-S060 × 9A, ICD-10-D-S060 × 9D, ICD-10-D-S060 × 9S, ICD-10-D-S061 × 0A, ICD-10-D-S061 × 0D, ICD-10-D-S061 × 0S, ICD-10-D-S061 × 1A, ICD-10-D-S061 × 1D, ICD-10-D-S061 × 1S, ICD-10-D-S061 × 2A, ICD-10-D-S061 × 2D, ICD-10-D-S061 × 2S, ICD-10-D-S061 × 3A, ICD-10-D-S061 × 3D, ICD-10-D-S061 × 3S, ICD-10-D-S061 × 4A, ICD-10-D-S061 × 4D, ICD-10-D-S061 × 4S, ICD-10-D-S061 × 5A, ICD-10-D-S061 × 5D, ICD-10-D-S061 × 5S, ICD-10-D-S061 × 6A, ICD-10-D-S061 × 6D, ICD-10-D-S061 × 6S, ICD-10-D-S061 × 7A, ICD-10-D-S061 × 7D, ICD-10-D-S061 × 7S, ICD-10-D-S061 × 8A, ICD-10-D-S061 × 8D, ICD-10-D-S061 × 8S, ICD-10-D-S061 × 9A, ICD-10-D-S061 × 9D, ICD-10-D-S061 × 9S, ICD-10-D-S062 × 0A, ICD-10-D-S062 × 0D, ICD-10-D-S062 × 0S, ICD-10-D-S062 × 1A, ICD-10-D-S062 × 1D, ICD-10-D-S062 × 1S, ICD-10-D-S062 × 2A, ICD-10-D-S062 × 2D, ICD-10-D-S062 × 2S, ICD-10-D-S062 × 3A, ICD-10-D-S062 × 3D, ICD-10-D-S062 × 3S, ICD-10-D-S062 × 4A, ICD-10-D-S062 × 4D, ICD-10-D-S062 × 4S, ICD-10-D-S062 × 5A, ICD-10-D-S062 × 5D, ICD-10-D-S062 × 5S, ICD-10-D-S062 × 6A, ICD-10-D-S062 × 6D, ICD-10-D-S062 × 6S, ICD-10-D-S062 × 7A, ICD-10-D-S062 × 7D, ICD-10-D-S062 × 7S, ICD-10-D-S062 × 8A, ICD-10-D-S062 × 8D, ICD-10-D-S062 × 8S, ICD-10-D-S062 × 9A, ICD-10-D-S062 × 9D, ICD-10-D-S062 × 9S, ICD-10-D-S06300A, ICD-10-D-S06300D, ICD-10-D-S06300S, ICD-10-D-S06301A, ICD-10-D-S06301D, ICD-10-D-S06301S, ICD-10-D-S06302A, ICD-10-D-S06302D, ICD-10-D-S06302S, ICD-10-D-S06303A, ICD-10-D-S06303D, ICD-10-D-S06303S, ICD-10-D-S06304A, ICD-10-D-S06304D, ICD-10-D-S06304S, ICD-10-D-S06305A, ICD-10-D-S06305D, ICD-10-D-S06305S, ICD-10-D-S06306A, ICD-10-D-S06306D, ICD-10-D-S06306S, ICD-10-D-S06307A, ICD-10-D-S06307D, ICD-10-D-S06307S, ICD-10-D-S06308A, ICD-10-D-S06308D, ICD-10-D-S06308S, ICD-10-D-S06309A, ICD-10-D-S06309D, ICD-10-D-S06309S, ICD-10-D-S06310A, ICD-10-D-S06310D, ICD-10-D-S06310S, ICD-10-D-S06311A, ICD-10-D-S06311D, ICD-10-D-S06311S, ICD-10-D-S06312A, ICD-10-D-S06312D, ICD-10-D-S06312S, ICD-10-D-S06313A, ICD-10-D-S06313D, ICD-10-D-S06313S, ICD-10-D-S06314A, ICD-10-D-S06314D, ICD-10-D-S06314S, ICD-10-D-S06315A, ICD-10-D-S06315D, ICD-10-D-S06315S, ICD-10-D-S06316A, ICD-10-D-S06316D, ICD-10-D-S06316S, ICD-10-D-S06317A, ICD-10-D-S06317D, ICD-10-D-S06317S, ICD-10-D-S06318A, ICD-10-D-S06318D, ICD-10-D-S06318S, ICD-10-D-S06319A, ICD-10-D-S06319D, ICD-10-D-S06319S, ICD-10-D-S06320A, ICD-10-D-S06320D, ICD-10-D-S06320S, ICD-10-D-S06321A, ICD-10-D-S06321D, ICD-10-D-S06321S, ICD-10-D-S06322A, ICD-10-D-S06322D, ICD-10-D-S06322S, ICD-10-D-S06323A, ICD-10-D-S06323D, ICD-10-D-S06323S, ICD-10-D-S06324A, ICD-10-D-S06324D, ICD-10-D-S06324S, ICD-10-D-S06325A, ICD-10-D-S06325D, ICD-10-D-S06325S, ICD-10-D-S06326A, ICD-10-D-S06326D, ICD-10-D-S06326S, ICD-10-D-S06327A, ICD-10-D-S06327D, ICD-10-D-S06327S, ICD-10-D-S06328A, ICD-10-D-S06328D, ICD-10-D-S06328S, ICD-10-D-S06329A, ICD-10-D-S06329D, ICD-10-D-S06329S, ICD-10-D-S06330A, ICD-10-D-S06330D, ICD-10-D-S06330S, ICD-10-D-S06331A, ICD-10-D-S06331D, ICD-10-D-S06331S, ICD-10-D-S06332A, ICD-10-D-S06332D, ICD-10-D-S06332S, ICD-10-D-S06333A, ICD-10-D-S06333D, ICD-10-D-S06333S, ICD-10-D-S06334A, ICD-10-D-S06334D, ICD-10-D-S06334S, ICD-10-D-S06335A, ICD-10-D-S06335D, ICD-10-D-S06335S, ICD-10-D-S06336A, ICD-10-D-S06336D, ICD-10-D-S06336S, ICD-10-D-S06337A, ICD-10-D-S06337D, ICD-10-D-S06337S, ICD-10-D-S06338A, ICD-10-D-S06338D, ICD-10-D-S06338S, ICD-10-D-S06339A, ICD-10-D-S06339D, ICD-10-D-S06339S, ICD-10-D-S06340A, ICD-10-D-S06340D, ICD-10-D-S06340S, ICD-10-D-S06341A, ICD-10-D-S06341D, ICD-10-D-S06341S, ICD-10-D-S06342A, ICD-10-D-S06342D, ICD-10-D-S06342S, ICD-10-D-S06343A, ICD-10-D-S06343D, ICD-10-D-S06343S, ICD-10-D-S06344A, ICD-10-D-S06344D, ICD-10-D-S06344S, ICD-10-D-S06345A, ICD-10-D-S06345D, ICD-10-D-S06345S, ICD-10-D-S06346A, ICD-10-D-S06346D, ICD-10-D-S06346S, ICD-10-D-S06347A, ICD-10-D-S06347D, ICD-10-D-S06347S, ICD-10-D-S06348A, ICD-10-D-S06348D, ICD-10-D-S06348S, ICD-10-D-S06349A, ICD-10-D-S06349D, ICD-10-D-S06349S, ICD-10-D-S06350A, ICD-10-D-S06350D, ICD-10-D-S06350S, ICD-10-D-S06351A, ICD-10-D-S06351D, ICD-10-D-S06351S, ICD-10-D-S06352A, ICD-10-D-S06352D, ICD-10-D-S06352S, ICD-10-D-S06353A, ICD-10-D-S06353D, ICD-10-D-S06353S, ICD-10-D-S06354A, ICD-10-D-S06354D, ICD-10-D-S06354S, ICD-10-D-S06355A, ICD-10-D-S06355D, ICD-10-D-S06355S, ICD-10-D-S06356A, ICD-10-D-S06356D, ICD-10-D-S06356S, ICD-10-D-S06357A, ICD-10-D-S06357D, ICD-10-D-S06357S, ICD-10-D-S06358A, ICD-10-D-S06358D, ICD-10-D-S06358S, ICD-10-D-S06359A, ICD-10-D-S06359D, ICD-10-D-S06359S, ICD-10-D-S06360A, ICD-10-D-S06360D, ICD-10-D-S06360S, ICD-10-D-S06361A, ICD-10-D-S06361D, ICD-10-D-S06361S, ICD-10-D-S06362A, ICD-10-D-S06362D, ICD-10-D-S06362S, ICD-10-D-S06363A, ICD-10-D-S06363D, ICD-10-D-S06363S, ICD-10-D-S06364A, ICD-10-D-S06364D, ICD-10-D-S06364S, ICD-10-D-S06365A, ICD-10-D-S06365D, ICD-10-D-S06365S, ICD-10-D-S06366A, ICD-10-D-S06366D, ICD-10-D-S06366S, ICD-10-D-S06367A, ICD-10-D-S06367D, ICD-10-D-S06367S, ICD-10-D-S06368A, ICD-10-D-S06368D, ICD-10-D-S06368S, ICD-10-D-S06369A, ICD-10-D-S06369D, ICD-10-D-S06369S, ICD-10-D-S06370A, ICD-10-D-S06370D, ICD-10-D-S06370S, ICD-10-D-S06371A, ICD-10-D-S06371D, ICD-10-D-S06371S, ICD-10-D-S06372A, ICD-10-D-S06372D, ICD-10-D-S06372S, ICD-10-D-S06373A, ICD-10-D-S06373D, ICD-10-D-S06373S, ICD-10-D-S06374A, ICD-10-D-S06374D, ICD-10-D-S06374S, ICD-10-D-S06375A, ICD-10-D-S06375D, ICD-10-D-S06375S, ICD-10-D-S06376A, ICD-10-D-S06376D, ICD-10-D-S06376S, ICD-10-D-S06377A, ICD-10-D-S06377D, ICD-10-D-S06377S, ICD-10-D-S06378A, ICD-10-D-S06378D, ICD-10-D-S06378S, ICD-10-D-S06379A, ICD-10-D-S06379D, ICD-10-D-S06379S, ICD-10-D-S06380A, ICD-10-D-S06380D, ICD-10-D-S06380S, ICD-10-D-S06381A, ICD-10-D-S06381D, ICD-10-D-S06381S, ICD-10-D-S06382A, ICD-10-D-S06382D, ICD-10-D-S06382S, ICD-10-D-S06383A, ICD-10-D-S06383D, ICD-10-D-S06383S, ICD-10-D-S06384A, ICD-10-D-S06384D, ICD-10-D-S06384S, ICD-10-D-S06385A, ICD-10-D-S06385D, ICD-10-D-S06385S, ICD-10-D-S06386A, ICD-10-D-S06386D, ICD-10-D-S06386S, ICD-10-D-S06387A, ICD-10-D-S06387D, ICD-10-D-S06387S, ICD-10-D-S06388A, ICD-10-D-S06388D, ICD-10-D-S06388S, ICD-10-D-S06389A, ICD-10-D-S06389D, ICD-10-D-S06389S, ICD-10-D-S064 × 0A, ICD-10-D-S064 × 0D, ICD-10-D-S064 × 0S, ICD-10-D-S064 × 1A, ICD-10-D-S064 × 1D, ICD-10-D-S064 × 1S, ICD-10-D-S064 × 2A, ICD-10-D-S064 × 2D, ICD-10-D-S064 × 2S, ICD-10-D-S064 × 3A, ICD-10-D-S064 × 3D, ICD-10-D-S064 × 3S, ICD-10-D-S064 × 4A, ICD-10-D-S064 × 4D, ICD-10-D-S064 × 4S, ICD-10-D-S064 × 5A, ICD-10-D-S064 × 5D, ICD-10-D-S064 × 5S, ICD-10-D-S064 × 6A, ICD-10-D-S064 × 6D, ICD-10-D-S064 × 6S, ICD-10-D-S064 × 7A, ICD-10-D-S064 × 7D, ICD-10-D-S064 × 7S, ICD-10-D-S064 × 8A, ICD-10-D-S064 × 8D, ICD-10-D-S064 × 8S, ICD-10-D-S064 × 9A, ICD-10-D-S064 × 9D, ICD-10-D-S064 × 9S, ICD-10-D-S065 × 0A, ICD-10-D-S065 × 0D, ICD-10-D-S065 × 0S, ICD-10-D-S065 × 1A, ICD-10-D-S065 × 1D, ICD-10-D-S065 × 1S, ICD-10-D-S065 × 2A, ICD-10-D-S065 × 2D, ICD-10-D-S065 × 2S, ICD-10-D-S065 × 3A, ICD-10-D-S065 × 3D, ICD-10-D-S065 × 3S, ICD-10-D-S065 × 4A, ICD-10-D-S065 × 4D, ICD-10-D-S065 × 4S, ICD-10-D-S065 × 5A, ICD-10-D-S065 × 5D, ICD-10-D-S065 × 5S, ICD-10-D-S065 × 6A, ICD-10-D-S065 × 6D, ICD-10-D-S065 × 6S, ICD-10-D-S065 × 7A, ICD-10-D-S065 × 7D, ICD-10-D-S065 × 7S, ICD-10-D-S065 × 8A, ICD-10-D-S065 × 8D, ICD-10-D-S065 × 8S, ICD-10-D-S065 × 9A, ICD-10-D-S065 × 9D, ICD-10-D-S065 × 9S, ICD-10-D-S066 × 0A, ICD-10-D-S066 × 0D, ICD-10-D-S066 × 0S, ICD-10-D-S066 × 1A, ICD-10-D-S066 × 1D, ICD-10-D-S066 × 1S, ICD-10-D-S066 × 2A, ICD-10-D-S066 × 2D, ICD-10-D-S066 × 2S, ICD-10-D-S066 × 3A, ICD-10-D-S066 × 3D, ICD-10-D-S066 × 3S, ICD-10-D-S066 × 4A, ICD-10-D-S066 × 4D, ICD-10-D-S066 × 4S, ICD-10-D-S066 × 5A, ICD-10-D-S066 × 5D, ICD-10-D-S066 × 5S, ICD-10-D-S066 × 6A, ICD-10-D-S066 × 6D, ICD-10-D-S066 × 6S, ICD-10-D-S066 × 7A, ICD-10-D-S066 × 7D, ICD-10-D-S066 × 7S, ICD-10-D-S066 × 8A, ICD-10-D-S066 × 8D, ICD-10-D-S066 × 8S, ICD-10-D-S066 × 9A, ICD-10-D-S066 × 9D, ICD-10-D-S066 × 9S, ICD-10-D-S06810A, ICD-10-D-S06810D, ICD-10-D-S06810S, ICD-10-D-S06811A, ICD-10-D-S06811D, ICD-10-D-S06811S, ICD-10-D-S06812A, ICD-10-D-S06812D, ICD-10-D-S06812S, ICD-10-D-S06813A, ICD-10-D-S06813D, ICD-10-D-S06813S, ICD-10-D-S06814A, ICD-10-D-S06814D, ICD-10-D-S06814S, ICD-10-D-S06815A, ICD-10-D-S06815D, ICD-10-D-S06815S, ICD-10-D-S06816A, ICD-10-D-S06816D, ICD-10-D-S06816S, ICD-10-D-S06817A, ICD-10-D-S06817D, ICD-10-D-S06817S, ICD-10-D-S06818A, ICD-10-D-S06818D, ICD-10-D-S06818S, ICD-10-D-S06819A, ICD-10-D-S06819D, ICD-10-D-S06819S, ICD-10-D-S06820A, ICD-10-D-S06820D, ICD-10-D-S06820S, ICD-10-D-S06821A, ICD-10-D-S06821D, ICD-10-D-S06821S, ICD-10-D-S06822A, ICD-10-D-S06822D, ICD-10-D-S06822S, ICD-10-D-S06823A, ICD-10-D-S06823D, ICD-10-D-S06823S, ICD-10-D-S06824A, ICD-10-D-S06824D, ICD-10-D-S06824S, ICD-10-D-S06825A, ICD-10-D-S06825D, ICD-10-D-S06825S, ICD-10-D-S06826A, ICD-10-D-S06826D, ICD-10-D-S06826S, ICD-10-D-S06827A, ICD-10-D-S06827D, ICD-10-D-S06827S, ICD-10-D-S06828A, ICD-10-D-S06828D, ICD-10-D-S06828S, ICD-10-D-S06829A, ICD-10-D-S06829D, ICD-10-D-S06829S, ICD-10-D-S06890A, ICD-10-D-S06890D, ICD-10-D-S06890S, ICD-10-D-S06891A, ICD-10-D-S06891D, ICD-10-D-S06891S, ICD-10-D-S06892A, ICD-10-D-S06892D, ICD-10-D-S06892S, ICD-10-D-S06893A, ICD-10-D-S06893D, ICD-10-D-S06893S, ICD-10-D-S06894A, ICD-10-D-S06894D, ICD-10-D-S06894S, ICD-10-D-S06895A, ICD-10-D-S06895D, ICD-10-D-S06895S, ICD-10-D-S06896A, ICD-10-D-S06896D, ICD-10-D-S06896S, ICD-10-D-S06897A, ICD-10-D-S06897D, ICD-10-D-S06897S, ICD-10-D-S06898A, ICD-10-D-S06898D, ICD-10-D-S06898S, ICD-10-D-S06899A, ICD-10-D-S06899D, ICD-10-D-S06899S, ICD-10-D-S069 × 0A, ICD-10-D-S069 × 0D, ICD-10-D-S069 × 0S, ICD-10-D-S069 × 1A, ICD-10-D-S069 × 1D, ICD-10-D-S069 × 1S, ICD-10-D-S069 × 2A, ICD-10-D-S069 × 2D, ICD-10-D-S069 × 2S, ICD-10-D-S069 × 3A, ICD-10-D-S069 × 3D, ICD-10-D-S069 × 3S, ICD-10-D-S069 × 4A, ICD-10-D-S069 × 4D, ICD-10-D-S069 × 4S, ICD-10-D-S069 × 5A, ICD-10-D-S069 × 5D, ICD-10-D-S069 × 5S, ICD-10-D-S069 × 6A, ICD-10-D-S069 × 6D, ICD-10-D-S069 × 6S, ICD-10-D-S069 × 7A, ICD-10-D-S069 × 7D, ICD-10-D-S069 × 7S, ICD-10-D-S069 × 8A, ICD-10-D-S069 × 8D, ICD-10-D-S069 × 8S, ICD-10-D-S069 × 9A, ICD-10-D-S069 × 9D, ICD-10-D-S069 × 9S, ICD-10-D-Z87820, ICD-9-D-8500, ICD-9-D-85011, ICD-9-D-85012, ICD-9-D-8502, ICD-9-D-8503, ICD-9-D-8504, ICD-9-D-8505, ICD-9-D-85141, ICD-9-D-85142, ICD-9-D-85143, ICD-9-D-85144, ICD-9-D-85145, ICD-9-D-85146, ICD-9-D-85181, ICD-9-D-85182, ICD-9-D-85183, ICD-9-D-85184, ICD-9-D-85185, ICD-9-D-85186, ICD-9-D-85201, ICD-9-D-85202, ICD-9-D-85203, ICD-9-D-85204, ICD-9-D-85205, ICD-9-D-85206, ICD-9-D-85221, ICD-9-D-85222, ICD-9-D-85223, ICD-9-D-85224, ICD-9-D-85225, ICD-9-D-85226, ICD-9-D-85241, ICD-9-D-85242, ICD-9-D-85243, ICD-9-D-85244, ICD-9-D-85245, ICD-9-D-85246, ICD-9-D-85301, ICD-9-D-85302, ICD-9-D-85303, ICD-9-D-85304, ICD-9-D-85305, ICD-9-D-85306, ICD-9-D-85401, ICD-9-D-85402, ICD-9-D-85403, ICD-9-D-85404, ICD-9-D-85405, ICD-9-D-85406, ICD-9-D-9070, ICD-9-D-V1552
Meningitis	ICD-10-D-A0101, ICD-10-D-A0221, ICD-10-D-A203, ICD-10-D-A2781, ICD-10-D-A3211, ICD-10-D-A390, ICD-10-D-A4281, ICD-10-D-A6921, ICD-10-D-A870, ICD-10-D-A871, ICD-10-D-A872, ICD-10-D-A878, ICD-10-D-A879, ICD-10-D-B003, ICD-10-D-B010, ICD-10-D-B021, ICD-10-D-B051, ICD-10-D-B0602, ICD-10-D-B261, ICD-10-D-B2702, ICD-10-D-B2712, ICD-10-D-B2782, ICD-10-D-B2792, ICD-10-D-B375, ICD-10-D-B384, ICD-10-D-B451, ICD-10-D-B5741, ICD-10-D-D8681, ICD-10-D-G000, ICD-10-D-G001, ICD-10-D-G002, ICD-10-D-G003, ICD-10-D-G008, ICD-10-D-G009, ICD-10-D-G01, ICD-10-D-G02, ICD-10-D-G030, ICD-10-D-G031, ICD-10-D-G032, ICD-10-D-G038, ICD-10-D-G039, ICD-9-D-10081, ICD-9-D-11283, ICD-9-D-1142, ICD-9-D-3200, ICD-9-D-3201, ICD-9-D-3202, ICD-9-D-3203, ICD-9-D-3207, ICD-9-D-32082, ICD-9-D-32089, ICD-9-D-321, ICD-9-D-3211, ICD-9-D-3220, ICD-9-D-3221, ICD-9-D-3222, ICD-9-D-3229, ICD-9-D-360, ICD-9-D-470, ICD-9-D-478, ICD-9-D-479, ICD-9-D-490, ICD-9-D-491, ICD-9-D-530, ICD-9-D-5472, ICD-9-D-721
Nervous system anomalies	ICD-10-D-G901, ICD-10-D-Q000, ICD-10-D-Q001, ICD-10-D-Q002, ICD-10-D-Q010, ICD-10-D-Q011, ICD-10-D-Q012, ICD-10-D-Q018, ICD-10-D-Q019, ICD-10-D-Q02, ICD-10-D-Q030, ICD-10-D-Q031, ICD-10-D-Q038, ICD-10-D-Q039, ICD-10-D-Q040, ICD-10-D-Q041, ICD-10-D-Q042, ICD-10-D-Q043, ICD-10-D-Q044, ICD-10-D-Q045, ICD-10-D-Q046, ICD-10-D-Q048, ICD-10-D-Q049, ICD-10-D-Q050, ICD-10-D-Q051, ICD-10-D-Q052, ICD-10-D-Q053, ICD-10-D-Q054, ICD-10-D-Q055, ICD-10-D-Q056, ICD-10-D-Q057, ICD-10-D-Q058, ICD-10-D-Q059, ICD-10-D-Q060, ICD-10-D-Q061, ICD-10-D-Q062, ICD-10-D-Q063, ICD-10-D-Q064, ICD-10-D-Q068, ICD-10-D-Q069, ICD-10-D-Q0700, ICD-10-D-Q0701, ICD-10-D-Q0702, ICD-10-D-Q0703, ICD-10-D-Q078, ICD-10-D-Q079, ICD-10-D-Q8500, ICD-10-D-Q8501, ICD-10-D-Q8502, ICD-10-D-Q8503, ICD-10-D-Q8509, ICD-10-D-Z87728, ICD-9-D-23770, ICD-9-D-23771, ICD-9-D-23772, ICD-9-D-23773, ICD-9-D-23779, ICD-9-D-7400, ICD-9-D-7401, ICD-9-D-7402, ICD-9-D-74100, ICD-9-D-74101, ICD-9-D-74102, ICD-9-D-74103, ICD-9-D-74190, ICD-9-D-74191, ICD-9-D-74192, ICD-9-D-74193, ICD-9-D-7424, ICD-9-D-74251, ICD-9-D-74253, ICD-9-D-74259, ICD-9-D-7428, ICD-9-D-7429, ICD-9-D-V1363
Other nervous system conditions	ICD-10-D-E7500, ICD-10-D-E7501, ICD-10-D-E7502, ICD-10-D-E7509, ICD-10-D-E7510, ICD-10-D-E7511, ICD-10-D-E7519, ICD-10-D-E7523, ICD-10-D-E7525, ICD-10-D-E7529, ICD-10-D-E754, ICD-10-D-G10, ICD-10-D-G110, ICD-10-D-G111, ICD-10-D-G112, ICD-10-D-G113, ICD-10-D-G114, ICD-10-D-G118, ICD-10-D-G119, ICD-10-D-G120, ICD-10-D-G121, ICD-10-D-G1220, ICD-10-D-G1221, ICD-10-D-G1222, ICD-10-D-G1223, ICD-10-D-G1224, ICD-10-D-G1225, ICD-10-D-G1229, ICD-10-D-G128, ICD-10-D-G129, ICD-10-D-G230, ICD-10-D-G231, ICD-10-D-G232, ICD-10-D-G238, ICD-10-D-G239, ICD-10-D-G2401, ICD-10-D-G2402, ICD-10-D-G2409, ICD-10-D-G241, ICD-10-D-G242, ICD-10-D-G243, ICD-10-D-G244, ICD-10-D-G245, ICD-10-D-G248, ICD-10-D-G249, ICD-10-D-G250, ICD-10-D-G251, ICD-10-D-G252, ICD-10-D-G253, ICD-10-D-G254, ICD-10-D-G255, ICD-10-D-G2561, ICD-10-D-G2569, ICD-10-D-G2570, ICD-10-D-G2571, ICD-10-D-G2579, ICD-10-D-G2581, ICD-10-D-G2582, ICD-10-D-G2583, ICD-10-D-G2589, ICD-10-D-G259, ICD-10-D-G26, ICD-10-D-G312, ICD-10-D-G3181, ICD-10-D-G3182, ICD-10-D-G3184, ICD-10-D-G3185, ICD-10-D-G3189, ICD-10-D-G319, ICD-10-D-G320, ICD-10-D-G3281, ICD-10-D-G803, ICD-10-D-G9001, ICD-10-D-G9009, ICD-10-D-G903, ICD-10-D-G904, ICD-10-D-G990, ICD-10-D-G992, ICD-9-D-3300, ICD-9-D-3301, ICD-9-D-3308, ICD-9-D-3309, ICD-9-D-3316, ICD-9-D-3317, ICD-9-D-33183, ICD-9-D-33189, ICD-9-D-3330, ICD-9-D-3331, ICD-9-D-3332, ICD-9-D-3333, ICD-9-D-3334, ICD-9-D-3335, ICD-9-D-3336, ICD-9-D-33371, ICD-9-D-33372, ICD-9-D-33379, ICD-9-D-33381, ICD-9-D-33382, ICD-9-D-33383, ICD-9-D-33384, ICD-9-D-33385, ICD-9-D-33389, ICD-9-D-33390, ICD-9-D-33391, ICD-9-D-33393, ICD-9-D-33394, ICD-9-D-33399, ICD-9-D-3340, ICD-9-D-3341, ICD-9-D-3342, ICD-9-D-3344, ICD-9-D-3348, ICD-9-D-3349, ICD-9-D-3350, ICD-9-D-33510, ICD-9-D-33511, ICD-9-D-33519, ICD-9-D-33520, ICD-9-D-33522, ICD-9-D-33524, ICD-9-D-33529, ICD-9-D-3362, ICD-9-D-3363, ICD-9-D-33700, ICD-9-D-33701, ICD-9-D-3371, ICD-9-D-3373
Other nervous system disorders	ICD-10-D-B2701, ICD-10-D-B2711, ICD-10-D-B2781, ICD-10-D-B2791, ICD-10-D-G08, ICD-10-D-G130, ICD-10-D-G131, ICD-10-D-G132, ICD-10-D-G138, ICD-10-D-G210, ICD-10-D-G2111, ICD-10-D-G2119, ICD-10-D-G212, ICD-10-D-G213, ICD-10-D-G214, ICD-10-D-G218, ICD-10-D-G219, ICD-10-D-G360, ICD-10-D-G361, ICD-10-D-G368, ICD-10-D-G369, ICD-10-D-G370, ICD-10-D-G371, ICD-10-D-G372, ICD-10-D-G373, ICD-10-D-G374, ICD-10-D-G375, ICD-10-D-G378, ICD-10-D-G379, ICD-10-D-G4720, ICD-10-D-G4721, ICD-10-D-G4722, ICD-10-D-G4723, ICD-10-D-G4724, ICD-10-D-G4725, ICD-10-D-G4726, ICD-10-D-G4727, ICD-10-D-G4729, ICD-10-D-G47411, ICD-10-D-G47419, ICD-10-D-G47421, ICD-10-D-G47429, ICD-10-D-G4763, ICD-10-D-G500, ICD-10-D-G501, ICD-10-D-G508, ICD-10-D-G509, ICD-10-D-G510, ICD-10-D-G511, ICD-10-D-G512, ICD-10-D-G513, ICD-10-D-G514, ICD-10-D-G518, ICD-10-D-G519, ICD-10-D-G520, ICD-10-D-G521, ICD-10-D-G522, ICD-10-D-G523, ICD-10-D-G527, ICD-10-D-G528, ICD-10-D-G529, ICD-10-D-G53, ICD-10-D-G540, ICD-10-D-G541, ICD-10-D-G542, ICD-10-D-G543, ICD-10-D-G544, ICD-10-D-G545, ICD-10-D-G546, ICD-10-D-G547, ICD-10-D-G548, ICD-10-D-G549, ICD-10-D-G55, ICD-10-D-G5600, ICD-10-D-G5601, ICD-10-D-G5602, ICD-10-D-G5603, ICD-10-D-G5610, ICD-10-D-G5611, ICD-10-D-G5612, ICD-10-D-G5613, ICD-10-D-G5620, ICD-10-D-G5621, ICD-10-D-G5622, ICD-10-D-G5623, ICD-10-D-G5630, ICD-10-D-G5631, ICD-10-D-G5632, ICD-10-D-G5633, ICD-10-D-G5640, ICD-10-D-G5641, ICD-10-D-G5642, ICD-10-D-G5643, ICD-10-D-G5680, ICD-10-D-G5681, ICD-10-D-G5682, ICD-10-D-G5683, ICD-10-D-G5690, ICD-10-D-G5691, ICD-10-D-G5692, ICD-10-D-G5693, ICD-10-D-G5700, ICD-10-D-G5701, ICD-10-D-G5702, ICD-10-D-G5703, ICD-10-D-G5710, ICD-10-D-G5711, ICD-10-D-G5712, ICD-10-D-G5713, ICD-10-D-G5720, ICD-10-D-G5721, ICD-10-D-G5722, ICD-10-D-G5723, ICD-10-D-G5730, ICD-10-D-G5731, ICD-10-D-G5732, ICD-10-D-G5733, ICD-10-D-G5740, ICD-10-D-G5741, ICD-10-D-G5742, ICD-10-D-G5743, ICD-10-D-G5750, ICD-10-D-G5751, ICD-10-D-G5752, ICD-10-D-G5753, ICD-10-D-G5760, ICD-10-D-G5761, ICD-10-D-G5762, ICD-10-D-G5763, ICD-10-D-G5770, ICD-10-D-G5771, ICD-10-D-G5772, ICD-10-D-G5773, ICD-10-D-G5780, ICD-10-D-G5781, ICD-10-D-G5782, ICD-10-D-G5783, ICD-10-D-G5790, ICD-10-D-G5791, ICD-10-D-G5792, ICD-10-D-G5793, ICD-10-D-G580, ICD-10-D-G587, ICD-10-D-G588, ICD-10-D-G589, ICD-10-D-G59, ICD-10-D-G600, ICD-10-D-G601, ICD-10-D-G602, ICD-10-D-G603, ICD-10-D-G608, ICD-10-D-G609, ICD-10-D-G610, ICD-10-D-G611, ICD-10-D-G6181, ICD-10-D-G6182, ICD-10-D-G6189, ICD-10-D-G619, ICD-10-D-G620, ICD-10-D-G622, ICD-10-D-G6281, ICD-10-D-G6282, ICD-10-D-G6289, ICD-10-D-G629, ICD-10-D-G63, ICD-10-D-G64, ICD-10-D-G650, ICD-10-D-G651, ICD-10-D-G652, ICD-10-D-G7000, ICD-10-D-G7001, ICD-10-D-G701, ICD-10-D-G702, ICD-10-D-G7080, ICD-10-D-G7081, ICD-10-D-G7089, ICD-10-D-G709, ICD-10-D-G710, ICD-10-D-G7111, ICD-10-D-G7112, ICD-10-D-G7113, ICD-10-D-G7114, ICD-10-D-G7119, ICD-10-D-G712, ICD-10-D-G713, ICD-10-D-G718, ICD-10-D-G719, ICD-10-D-G720, ICD-10-D-G721, ICD-10-D-G722, ICD-10-D-G723, ICD-10-D-G7241, ICD-10-D-G7249, ICD-10-D-G7281, ICD-10-D-G7289, ICD-10-D-G729, ICD-10-D-G731, ICD-10-D-G733, ICD-10-D-G737, ICD-10-D-G890, ICD-10-D-G8911, ICD-10-D-G8912, ICD-10-D-G8918, ICD-10-D-G8921, ICD-10-D-G8922, ICD-10-D-G8928, ICD-10-D-G8929, ICD-10-D-G893, ICD-10-D-G894, ICD-10-D-G902, ICD-10-D-G9050, ICD-10-D-G90511, ICD-10-D-G90512, ICD-10-D-G90513, ICD-10-D-G90519, ICD-10-D-G90521, ICD-10-D-G90522, ICD-10-D-G90523, ICD-10-D-G90529, ICD-10-D-G9059, ICD-10-D-G908, ICD-10-D-G909, ICD-10-D-G910, ICD-10-D-G911, ICD-10-D-G912, ICD-10-D-G913, ICD-10-D-G914, ICD-10-D-G918, ICD-10-D-G919, ICD-10-D-G92, ICD-10-D-G930, ICD-10-D-G932, ICD-10-D-G9340, ICD-10-D-G9341, ICD-10-D-G9349, ICD-10-D-G935, ICD-10-D-G936, ICD-10-D-G937, ICD-10-D-G9381, ICD-10-D-G9382, ICD-10-D-G9389, ICD-10-D-G939, ICD-10-D-G94, ICD-10-D-G950, ICD-10-D-G9511, ICD-10-D-G9519, ICD-10-D-G9520, ICD-10-D-G9529, ICD-10-D-G9581, ICD-10-D-G9589, ICD-10-D-G959, ICD-10-D-G960, ICD-10-D-G9612, ICD-10-D-G9619, ICD-10-D-G968, ICD-10-D-G969, ICD-10-D-G980, ICD-10-D-G988, ICD-10-D-G998, ICD-10-D-J1081, ICD-10-D-R200, ICD-10-D-R201, ICD-10-D-R202, ICD-10-D-R203, ICD-10-D-R208, ICD-10-D-R209, ICD-10-D-R250, ICD-10-D-R251, ICD-10-D-R253, ICD-10-D-R258, ICD-10-D-R259, ICD-10-D-R260, ICD-10-D-R261, ICD-10-D-R262, ICD-10-D-R2681, ICD-10-D-R2689, ICD-10-D-R269, ICD-10-D-R270, ICD-10-D-R278, ICD-10-D-R279, ICD-10-D-R290, ICD-10-D-R292, ICD-10-D-R414, ICD-10-D-R41840, ICD-10-D-R41841, ICD-10-D-R41842, ICD-10-D-R41843, ICD-10-D-R41844, ICD-10-D-R4189, ICD-10-D-R430, ICD-10-D-R431, ICD-10-D-R432, ICD-10-D-R438, ICD-10-D-R439, ICD-10-D-R4701, ICD-10-D-R4702, ICD-10-D-R471, ICD-10-D-R4781, ICD-10-D-R4782, ICD-10-D-R4789, ICD-10-D-R479, ICD-10-D-R481, ICD-10-D-R482, ICD-10-D-R488, ICD-10-D-R489, ICD-10-D-R900, ICD-10-D-R9082, ICD-10-D-Z462, ICD-10-D-Z86011, ICD-10-D-Z8661, ICD-10-D-Z8669, ICD-10-D-Z982, ICD-9-D-32371, ICD-9-D-325, ICD-9-D-32730, ICD-9-D-32731, ICD-9-D-32732, ICD-9-D-32733, ICD-9-D-32734, ICD-9-D-32735, ICD-9-D-32736, ICD-9-D-32737, ICD-9-D-32739, ICD-9-D-32753, ICD-9-D-3313, ICD-9-D-3314, ICD-9-D-3315, ICD-9-D-33181, ICD-9-D-3321, ICD-9-D-33392, ICD-9-D-3360, ICD-9-D-3361, ICD-9-D-3368, ICD-9-D-3369, ICD-9-D-33720, ICD-9-D-33721, ICD-9-D-33722, ICD-9-D-33729, ICD-9-D-3379, ICD-9-D-3380, ICD-9-D-33811, ICD-9-D-33812, ICD-9-D-33818, ICD-9-D-33821, ICD-9-D-33822, ICD-9-D-33828, ICD-9-D-33829, ICD-9-D-3383, ICD-9-D-3384, ICD-9-D-3410, ICD-9-D-3411, ICD-9-D-34120, ICD-9-D-3418, ICD-9-D-3419, ICD-9-D-34700, ICD-9-D-34701, ICD-9-D-34710, ICD-9-D-34711, ICD-9-D-3480, ICD-9-D-3482, ICD-9-D-34830, ICD-9-D-34831, ICD-9-D-34839, ICD-9-D-3484, ICD-9-D-3485, ICD-9-D-34881, ICD-9-D-34882, ICD-9-D-34889, ICD-9-D-3489, ICD-9-D-3492, ICD-9-D-34981, ICD-9-D-3499, ICD-9-D-3501, ICD-9-D-3502, ICD-9-D-3508, ICD-9-D-3509, ICD-9-D-3510, ICD-9-D-3511, ICD-9-D-3518, ICD-9-D-3519, ICD-9-D-3520, ICD-9-D-3521, ICD-9-D-3523, ICD-9-D-3524, ICD-9-D-3525, ICD-9-D-3526, ICD-9-D-3529, ICD-9-D-3530, ICD-9-D-3531, ICD-9-D-3532, ICD-9-D-3533, ICD-9-D-3534, ICD-9-D-3535, ICD-9-D-3536, ICD-9-D-3538, ICD-9-D-3539, ICD-9-D-3540, ICD-9-D-3541, ICD-9-D-3542, ICD-9-D-3543, ICD-9-D-3544, ICD-9-D-3545, ICD-9-D-3548, ICD-9-D-3549, ICD-9-D-3550, ICD-9-D-3551, ICD-9-D-3552, ICD-9-D-3553, ICD-9-D-3554, ICD-9-D-3555, ICD-9-D-3556, ICD-9-D-35571, ICD-9-D-35579, ICD-9-D-3558, ICD-9-D-3559, ICD-9-D-3560, ICD-9-D-3563, ICD-9-D-3564, ICD-9-D-3568, ICD-9-D-3569, ICD-9-D-3570, ICD-9-D-3573, ICD-9-D-3574, ICD-9-D-3576, ICD-9-D-3577, ICD-9-D-35781, ICD-9-D-35782, ICD-9-D-35789, ICD-9-D-3579, ICD-9-D-35800, ICD-9-D-35801, ICD-9-D-3581, ICD-9-D-3582, ICD-9-D-35830, ICD-9-D-35831, ICD-9-D-35839, ICD-9-D-3588, ICD-9-D-3589, ICD-9-D-3590, ICD-9-D-3591, ICD-9-D-35921, ICD-9-D-35922, ICD-9-D-35923, ICD-9-D-35924, ICD-9-D-35929, ICD-9-D-3593, ICD-9-D-3594, ICD-9-D-3595, ICD-9-D-35971, ICD-9-D-35979, ICD-9-D-35981, ICD-9-D-35989, ICD-9-D-3599, ICD-9-D-7197, ICD-9-D-7813, ICD-9-D-7817, ICD-9-D-7818, ICD-9-D-7820, ICD-9-D-7842, ICD-9-D-7843, ICD-9-D-78451, ICD-9-D-78452, ICD-9-D-78459, ICD-9-D-78460, ICD-9-D-78469, ICD-9-D-7930, ICD-9-D-7961, ICD-9-D-79951, ICD-9-D-79952, ICD-9-D-79953, ICD-9-D-79954, ICD-9-D-79955, ICD-9-D-79959, ICD-9-D-V1240, ICD-9-D-V1241, ICD-9-D-V1242, ICD-9-D-V452, ICD-9-D-V5309
Other central nervous system	ICD-10-D-A800, ICD-10-D-A801, ICD-10-D-A802, ICD-10-D-A8030, ICD-10-D-A8039, ICD-10-D-A804, ICD-10-D-A809, ICD-10-D-A8100, ICD-10-D-A8101, ICD-10-D-A8109, ICD-10-D-A812, ICD-10-D-A8181, ICD-10-D-A8182, ICD-10-D-A8183, ICD-10-D-A8189, ICD-10-D-A819, ICD-10-D-A880, ICD-10-D-A881, ICD-10-D-A888, ICD-10-D-B91, ICD-10-D-G060, ICD-10-D-G061, ICD-10-D-G062, ICD-10-D-G07, ICD-10-D-G09, ICD-10-D-G14, ICD-10-D-I673, ICD-10-D-Z8612, ICD-9-D-138, ICD-9-D-3240, ICD-9-D-3241, ICD-9-D-3249, ICD-9-D-326, ICD-9-D-4510, ICD-9-D-4520, ICD-9-D-4590, ICD-9-D-460, ICD-9-D-4611, ICD-9-D-4619, ICD-9-D-463, ICD-9-D-4671, ICD-9-D-4672, ICD-9-D-4679, ICD-9-D-469, ICD-9-D-48, ICD-9-D-7881, ICD-9-D-V1202

^a^The study cohort was limited to patients ≤25 years old with or without a diagnosis of attention-deficit/hyperactivity disorder prior to anterior cruciate ligament (ACL) reconstruction surgery who had claims available for at least 2 years prior to ACL reconstruction and 2 years postoperatively. The study period was 2010 to 2023.

## Appendix 2. Comorbidity Definitions

**Table:**

Comorbidity	*International Classification of Diseases, Ninth Revision* (ICD-9) and *International Classification of Diseases, Tenth Revision* (ICD-10) Codes
Alcohol use disorders	ICD-10-D-F1010, ICD-10-D-F1011, ICD-10-D-F10120, ICD-10-D-F10121, ICD-10-D-F10129, ICD-10-D-F1014, ICD-10-D-F10150, ICD-10-D-F10159, ICD-10-D-F10180, ICD-10-D-F10188, ICD-10-D-F1019, ICD-10-D-F1020, ICD-10-D-F1021, ICD-10-D-F10220, ICD-10-D-F10221, ICD-10-D-F10229, ICD-10-D-F10230, ICD-10-D-F10231, ICD-10-D-F10232, ICD-10-D-F10239, ICD-10-D-F1024, ICD-10-D-F10251, ICD-10-D-F10259, ICD-10-D-F1027, ICD-10-D-F10282, ICD-10-D-F10288, ICD-10-D-F1029, ICD-10-D-F10920, ICD-10-D-F10921, ICD-10-D-F10929, ICD-10-D-F10959, ICD-10-D-F10980, ICD-10-D-F1099, ICD-10-D-F3130, ICD-10-D-F3131, ICD-10-D-F3132, ICD-10-D-F314, ICD-10-D-F319, ICD-10-D-F320, ICD-10-D-F321, ICD-10-D-F322, ICD-10-D-F323, ICD-10-D-F329, ICD-10-D-F330, ICD-10-D-F331, ICD-10-D-F332, ICD-10-D-F333, ICD-10-D-F3341, ICD-10-D-F339, ICD-10-D-F341, ICD-10-D-F39, ICD-10-D-F4010, ICD-10-D-F410, ICD-10-D-F411, ICD-10-D-F418, ICD-10-D-F419, ICD-10-D-F430, ICD-10-D-F4310, ICD-10-D-F4312, ICD-10-D-G621, ICD-10-D-I426, ICD-10-D-K2920, ICD-10-D-K2921, ICD-10-D-K700, ICD-10-D-K7010, ICD-10-D-K7011, ICD-10-D-K7030, ICD-10-D-K7031, ICD-10-D-K7040, ICD-10-D-K709, ICD-10-D-M23611, ICD-10-D-M23612, ICD-10-D-O99311, ICD-10-D-O99314, ICD-10-D-Q860, ICD-10-D-S83501A, ICD-10-D-S83502A, ICD-10-D-S83511A, ICD-10-D-S83511D, ICD-10-D-S83511S, ICD-10-D-S83512A, ICD-10-D-S83512D, ICD-10-D-S83512S, ICD-9-D-2910, ICD-9-D-2914, ICD-9-D-29181, ICD-9-D-30300, ICD-9-D-30390, ICD-9-D-30500, ICD-9-D-4255, ICD-9-D-53530, ICD-9-D-5710, ICD-9-D-64844, ICD-9-D-76071
Obesity	ICD-10-D-E660, ICD-10-D-E669, ICD-9-D-2780, ICD-9-D-27800, ICD-9-D-27801, ICD-9-D-27802, ICD-9-D-27803
Tobacco use	ICD-10-D-F17200, ICD-10-D-F17201, ICD-10-D-F17203, ICD-10-D-F17208, ICD-10-D-F17209, ICD-10-D-F17210, ICD-10-D-F17211, ICD-10-D-F17213, ICD-10-D-F17218, ICD-10-D-F17219, ICD-10-D-F17220, ICD-10-D-F17220, ICD-10-D-F17221, ICD-10-D-F17221, ICD-10-D-F17223, ICD-10-D-F17223, ICD-10-D-F17228, ICD-10-D-F17228, ICD-10-D-F17229, ICD-10-D-F17229, ICD-10-D-F17290, ICD-10-D-F17290, ICD-10-D-F17291, ICD-10-D-F17291, ICD-10-D-F17293, ICD-10-D-F17293, ICD-10-D-F17298, ICD-10-D-F17298, ICD-10-D-F17299, ICD-10-D-F17299, ICD-10-D-Z716, ICD-10-D-Z720, ICD-10-D-Z720, ICD-10-D-Z87891, ICD-9-D-3051, ICD-9-D-98984, ICD-9-D-V1582
Anxiety disorders/depression	Anxiety disorders: ICD-10-D-F064, ICD-10-D-F4000, ICD-10-D-F4001, ICD-10-D-F4002, ICD-10-D-F4010, ICD-10-D-F4011, ICD-10-D-F40210, ICD-10-D-F40218, ICD-10-D-F40220, ICD-10-D-F40228, ICD-10-D-F40230, ICD-10-D-F40231, ICD-10-D-F40232, ICD-10-D-F40233, ICD-10-D-F40240, ICD-10-D-F40241, ICD-10-D-F40242, ICD-10-D-F40243, ICD-10-D-F40248, ICD-10-D-F40290, ICD-10-D-F40291, ICD-10-D-F40298, ICD-10-D-F408, ICD-10-D-F409, ICD-10-D-F410, ICD-10-D-F411, ICD-10-D-F413, ICD-10-D-F418, ICD-10-D-F419, ICD-10-D-F42, ICD-10-D-F422, ICD-10-D-F423, ICD-10-D-F424, ICD-10-D-F428, ICD-10-D-F429, ICD-10-D-F430, ICD-10-D-F4310, ICD-10-D-F4311, ICD-10-D-F4312, ICD-10-D-F488, ICD-10-D-F489, ICD-10-D-R452, ICD-10-D-R453, ICD-10-D-R454, ICD-10-D-R455, ICD-10-D-R456, ICD-10-D-R457, ICD-10-D-R4581, ICD-10-D-R4582, ICD-10-D-R4583, ICD-10-D-R4584, ICD-9-D-29384, ICD-9-D-30000, ICD-9-D-30001, ICD-9-D-30002, ICD-9-D-30009, ICD-9-D-30020, ICD-9-D-30021, ICD-9-D-30022, ICD-9-D-30023, ICD-9-D-30029, ICD-9-D-3003, ICD-9-D-3005, ICD-9-D-3009, ICD-9-D-3089, ICD-9-D-30981, ICD-9-D-78095, ICD-9-D-79922, ICD-9-D-79925 Depression: ICD-10-D-F3130, ICD-10-D-F3131, ICD-10-D-F3132, ICD-10-D-F314, ICD-10-D-F315, ICD-10-D-F3160, ICD-10-D-F3161, ICD-10-D-F3162, ICD-10-D-F3163, ICD-10-D-F3164, ICD-10-D-F319, ICD-10-D-F320, ICD-10-D-F321, ICD-10-D-F322, ICD-10-D-F323, ICD-10-D-F324, ICD-10-D-F325, ICD-10-D-F328, ICD-10-D-F329, ICD-10-D-F329, ICD-10-D-F330, ICD-10-D-F331, ICD-10-D-F332, ICD-10-D-F333, ICD-10-D-F3341, ICD-10-D-F3342, ICD-10-D-F338, ICD-10-D-F339, ICD-10-D-F341, ICD-10-D-F348, ICD-10-D-F349, ICD-10-D-F380, ICD-10-D-F381, ICD-10-D-F388, ICD-10-D-F39, ICD-10-D-F412, ICD-10-D-F432, ICD-9-D-2962, ICD-9-D-29621, ICD-9-D-29622, ICD-9-D-29623, ICD-9-D-29624, ICD-9-D-29625, ICD-9-D-29626, ICD-9-D-2963, ICD-9-D-29631, ICD-9-D-29632, ICD-9-D-29633, ICD-9-D-29634, ICD-9-D-29635, ICD-9-D-29636, ICD-9-D-29651, ICD-9-D-29652, ICD-9-D-29653, ICD-9-D-29654, ICD-9-D-29682, ICD-9-D-29682, ICD-9-D-2969, ICD-9-D-29699, ICD-9-D-298, ICD-9-D-3004, ICD-9-D-3090, ICD-9-D-3091, ICD-9-D-30928, ICD-9-D-311
Developmental disorders	ICD-10-D-F70, ICD-10-D-F71, ICD-10-D-F72, ICD-10-D-F73, ICD-10-D-F78, ICD-10-D-F79, ICD-10-D-F800, ICD-10-D-F801, ICD-10-D-F802, ICD-10-D-F804, ICD-10-D-F8081, ICD-10-D-F8082, ICD-10-D-F8089, ICD-10-D-F809, ICD-10-D-F810, ICD-10-D-F812, ICD-10-D-F8181, ICD-10-D-F8189, ICD-10-D-F819, ICD-10-D-F82, ICD-10-D-F88, ICD-10-D-F89, ICD-10-D-F985, ICD-10-D-R4183, ICD-10-D-R480, ICD-9-D-3070, ICD-9-D-31500, ICD-9-D-31501, ICD-9-D-31509, ICD-9-D-3151, ICD-9-D-3152, ICD-9-D-31531, ICD-9-D-31534, ICD-9-D-31535, ICD-9-D-31539, ICD-9-D-3154, ICD-9-D-3158, ICD-9-D-3159, ICD-9-D-317, ICD-9-D-3180, ICD-9-D-3181, ICD-9-D-3182

## Appendix 3. Outcome Measures and Medication Definitions

**Table:**

Outcome/Medication	*International Classification of Diseases, Ninth Revision* (ICD-9), *International Classification of Diseases, Tenth Revision* (ICD-10), Current Procedural Terminology (CPT), and Uniform System of Classification (USC) Codes and Standardized Drug Identifiers
Outcomes	
Repeat anterior cruciate ligament reconstruction	CPT-29888
Other knee surgery	Lysis of adhesions: CPT-29884 Total knee arthroplasty: CPT-27447 Medial collateral ligament/posterior cruciate ligament repair/reconstruction: CPT-27407, CPT-27427, CPT-27428, CPT-27429, CPT-27405, CPT-29889, ICD-10-D-M23621, ICD-10-D-M23622, ICD-10-D-M23629, ICD-10-D-S83521A, ICD-10-D-S83521D, ICD-10-D-S83521S, ICD-10-D-S83522A, ICD-10-D-S83522D, ICD-10-D-S83522S, ICD-10-D-S83529A, ICD-10-D-S83529D, ICD-10-D-S83529S, ICD-9-D-8440, ICD-9-D-8441 Meniscus repair/reconstruction: CPT-29880, CPT-29881, CPT-29882, CPT-29883
Stiffness	ICD-9-D-71846, ICD-9-D-71856, CPT-27570, ICD-10-D-M24562, ICD-10-D-M24569, ICD-10-P-9WB6XLZ, ICD-10-D-M25661, ICD-10-D-M25662, ICD-10-D-M25669
Opioid use^a^	USC-02211, USC-02212, USC-02214, USC-02221, USC-02222, USC-02232
Medications	
Stimulants	DRUG-METHYLPHENIDATE, DRUG-METHYLPHENIDATE_ER, DRUG-METHYLPHENIDATE_HCL, DRUG-METHYLPHENIDATE_HCL_CD, DRUG-METHYLPHENIDATE_HCL_ER, DRUG-METHYLPHENIDATE_LA, DRUG-METHYLPHENIDATE_SR, GENERIC_DRUG-DEXMETHYLPHENIDATE_HCL, GENERIC_DRUG-METHYLPHENIDATE, GENERIC_DRUG-METHYLPHENIDATE_HCL, DRUG-CONCERTA, DRUG-DAYTRANA, DRUG-DESOXYN, DRUG-METADATE_CD, DRUG-METADATE_ER, DRUG-METHYLIN, DRUG-METHYLIN_ER, DRUG-RITALIN, DRUG-RITALIN-SR, DRUG-RITALIN_LA, DRUG-DEXMETHYLPHENIDATE_HCL, DRUG-DEXMETHYLPHENIDATE_HCL_ER, DRUG-FOCALIN, DRUG-FOCALIN_XR, DRUG-AMPHETAMINE, DRUG-AMPHETAMINE_SULFATE, DRUG-DEXTROAMPHETAMINE-AMPHETAMINE, DRUG-DEXTROAMPHETAMINE-AMPHET_ER, DRUG-DEXTROAMPHETAMINE_SULFATE, DRUG-DEXTROAMPHETAMINE_SULFATE_ER, DRUG-HYDROXYAMPHETAMINE_HBR, DRUG-METHAMPHETAMINE_HCL, GENERIC_DRUG-AMPHETAMINE, GENERIC_DRUG-AMPHETAMINE_SULFATE, GENERIC_DRUG-DEXTROAMPHETAMINE/AMPHETAMINE, GENERIC_DRUG-DEXTROAMPHETAMINE_SULFATE, GENERIC_DRUG-HYDROXYAMPHETAMINE/TROPICAMIDE, GENERIC_DRUG-HYDROXYAMPHETAMINE_HBR, GENERIC_DRUG-METHAMPHETAMINE_HCL, DRUG-ADDERALL, DRUG-ADDERALL_XR, DRUG-DEXEDRINE, DRUG-VYVANSE, GENERIC_DRUG-LISDEXAMFETAMINE_DIMESYLATE
Nonstimulants	DRUG-NOREPINEPHRINE_BITARTRATE, DRUG-NOREPINEPHRINE_BITARTRATE-D5W, GENERIC_DRUG-NOREPINEPHRINE_BITARTRATE, GENERIC_DRUG-NOREPINEPHRINE_BITARTRATE/D5W, DRUG-ATOMOXETINE_HCL, DRUG-STRATTERA, GENERIC_DRUG-ATOMOXETINE_HCL
Agonists	DRUG-APRACLONIDINE_HCL, DRUG-CLONIDINE, DRUG-CLONIDINE_HCL, DRUG-CLONIDINE_HCL_ER, DRUG-GUANFACINE_HCL, DRUG-GUANFACINE_HCL_ER, DRUG-GUANFACINE_HYDROCHLORIDE, GENERIC_DRUG-APRACLONIDINE_HCL, GENERIC_DRUG-CLONIDINE, GENERIC_DRUG-CLONIDINE_HCL, GENERIC_DRUG-CLONIDINE_HCL/CHLORTHALIDONE, GENERIC_DRUG-CLONIDINE_HCL/PF, GENERIC_DRUG-GUANFACINE_HCL
Other drugs^b^	DRUG-APLENZIN, DRUG-WELLBUTRIN, DRUG-WELLBUTRIN_SR, DRUG-WELLBUTRIN_XL, DRUG-ZYBAN, DRUG-IMIPRAMINE_HCL, DRUG-IMIPRAMINE_PAMOATE, DRUG-TRIMIPRAMINE_MALEATE, DRUG-TOFRANIL, DRUG-TOFRANIL-PM, DRUG-CARBAMAZEPINE, DRUG-CARBAMAZEPINE_ER, GENERIC_DRUG-CARBAMAZEPINE, DRUG-EQUETRO, DRUG-TEGRETOL, DRUG-TEGRETOL_XR, DRUG-CLONAZEPAM, GENERIC_DRUG-CLONAZEPAM, DRUG-KLONOPIN, DRUG-DIVALPROEX_SODIUM, DRUG-DIVALPROEX_SODIUM_ER, GENERIC_DRUG-DIVALPROEX_SODIUM, DRUG-DEPAKOTE, DRUG-DEPAKOTE_ER, DRUG-DEPAKOTE_SPRINKLE, DRUG-LITHIUM, DRUG-LITHIUM_CARBONATE, DRUG-LITHIUM_CARBONATE_ER, DRUG-LITHIUM_CITRATE, DRUG-LITHIUM_CITRATE_TETRAHYDRATE, GENERIC_DRUG-LITHIUM_ASPARTATE, GENERIC_DRUG-LITHIUM_CARBONATE, GENERIC_DRUG-LITHIUM_CITRATE, GENERIC_DRUG-LITHIUM_CITRATE_TETRAHYDRATE, DRUG-ESKALITH, DRUG-ESKALITH_CR, DRUG-LITHOBID, DRUG-OLANZAPINE, DRUG-OLANZAPINE-FLUOXETINE_HCL, DRUG-OLANZAPINE_ODT, GENERIC_DRUG-OLANZAPINE, GENERIC_DRUG-OLANZAPINE/FLUOXETINE_HCL, GENERIC_DRUG-OLANZAPINE/SAMIDORPHAN_MALATE, GENERIC_DRUG-OLANZAPINE_PAMOATE, DRUG-ZYPREXA, DRUG-ZYPREXA_RELPREVV, DRUG-ZYPREXA_ZYDIS, DRUG-PAROXETINE_CR, DRUG-PAROXETINE_ER, DRUG-PAROXETINE_HCL, DRUG-PAROXETINE_MESYLATE, GENERIC_DRUG-PAROXETINE_HCL, GENERIC_DRUG-PAROXETINE_MESYLATE, DRUG-BRISDELLE, DRUG-PAXIL, DRUG-PAXIL_CR, DRUG-PEXEVA, DRUG-RISPERIDONE, DRUG-RISPERIDONE_ODT, GENERIC_DRUG-RISPERIDONE, GENERIC_DRUG-RISPERIDONE_MICROSPHERES, DRUG-RISPERDAL, DRUG-RISPERDAL_CONSTA, DRUG-RISPERDAL_M-TAB, DRUG-SERTRALINE_HCL, GENERIC_DRUG-SERTRALINE_HCL, DRUG-FLUOXETINE_DR, DRUG-FLUOXETINE_HCL, DRUG-OLANZAPINE-FLUOXETINE_HCL, GENERIC_DRUG-FLUOXETINE, GENERIC_DRUG-FLUOXETINE_HCL, GENERIC_DRUG-FLUOXETINE_HCL/DIET.SUPP_NO.17, GENERIC_DRUG-OLANZAPINE/FLUOXETINE_HCL, DRUG-PROZAC, DRUG-PROZAC_WEEKLY, DRUG-SARAFEM, DRUG-HYDROXYZINE_HCL, DRUG-HYDROXYZINE_PAMOATE, GENERIC_DRUG-HYDROXYZINE_HCL, GENERIC_DRUG-HYDROXYZINE_PAMOATE, GENERIC_DRUG-THEOPHYLLINE/EPHED/HYDROXYZINE, DRUG-ATARAX, DRUG-VISTARIL, DRUG-SERTRALINE_HCL, DRUG-ZOLOFT, DRUG-TRAZODONE, DRUG-TRAZODONE_HCL, GENERIC_DRUG-TRAZODONE_HCL, GENERIC_DRUG-TRAZODONE_HCL/DIET8, DRUG-DESYREL, DRUG-OLEPTRO_ER

^a^Opioid use was defined as prolonged use continuing at 2 years postoperatively.

^b^The other drugs category includes norepinephrine and dopamine reuptake inhibitors, serotonin and norepinephrine reuptake inhibitors, anticonvulsants, lithium, antipsychotics, selective serotonin reuptakle inhibitors, clonazepam, hydroxyzine, and trazodone.
